# Classification of avulsion fractures of the fifth metatarsal base using three-dimensional CT mapping and anatomical assessment: a retrospective case series study

**DOI:** 10.1186/s13047-022-00571-2

**Published:** 2022-08-31

**Authors:** Wenbao He, Haichao Zhou, Yingqi Zhang, Tao Yu, Jiang Xia, Youguang Zhao, Yunfeng Yang, Bing Li

**Affiliations:** Department of Orthopedics, Shanghai Tongji Hospital, School of Medicine, Tongji University, Shanghai, 200065 China

**Keywords:** Fifth metatarsal bone fractures, 3D mapping, Fracture classification, Anatomy, Injury mechanism

## Abstract

**Background:**

To clarify the injury mechanism of the avulsion fracture of the fifth metatarsal combining 3-dimensional (3D) fracture mapping with anatomical measurements.

**Methods:**

Two hundred twenty-two patients with the avulsion fractures of the fifth metatarsal base, who were admitted to our hospital from August 2015 to August 2020. The computed tomography (CT) scans were used to generate the 3-D images of all mapped fracture lines for the avulsion fractures of the fifth metatarsal base were compiled in an overall 3D image. The fifth metatarsal base of 8 unpaired lower limbs of adult Asian frozen cadaveric specimens were also dissected to observe and measure the specific locations of the attachment points of the peroneus brevis, lateral band of the plantar fascia, and peroneus tertius to the fifth metatarsal base.

**Results:**

Based on the type of fracture line produced and the specific locations of the attachment points of the tendons or fascia, the avulsion fractures of the fifth metatarsal base can be classified into three types: type I predominantly involves the action of the lateral band of the plantar fascia; type II predominantly involves the action of the peroneus brevis; type IIIA involves the joint action of the peroneus brevis and lateral band of the plantar fascia with one fracture line, and type IIIB involves the joint action of the peroneus brevis and lateral band of the plantar fascia with two fracture lines.

**Conclusion:**

The lateral band of the plantar fascia and peroneus brevis play a major role, either separately or together, in avulsion fractures of the fifth metatarsal base. With this knowledge, we propose a novel classification based on the injury mechanism, which can serve as a reference for clinical treatment and diagnosis.

**Level of evidence:**

Level III, retrospective case series.

## Background

Metatarsal fractures occur in approximately 67/100,000 individuals each year, 70% of which are fifth metatarsal fractures, while avulsion fractures account for two-thirds of all fifth metatarsal fractures [1–3]. Without treatment, it can lead to sequelae such as intractable pain and metatarsalgia [4]. The base of the fifth metatarsal bone is attached to several tendons and ligaments, including the peroneus brevis, the peroneus tertius, and the lateral band of the plantar fascia [5–7]. When the foot is subjected to an abnormal force, resulting in hindfoot inversion and forefoot plantarflexion, the unique anatomical structure of the fifth metatarsal bone directs the force to the proximal base of the bone, which is susceptible to avulsion fractures of the base.

Clinically, avulsion fractures of the fifth metatarsal base exhibit a diverse range of fracture morphologies, which have been beyond the description of existing classifications, but research on their fracture line characteristics is limited [8–11]. In addition, it is difficult for clinicians to choose treatment or predict prognosis with existing classifications for some special types. Therefore, in this study, we investigated the three-dimensional distribution of avulsion fractures of the fifth metatarsal base and conducted an anatomical study on the attachment site locations for the lateral band of the plantar fascia, peroneus brevis, and the peroneus tertius on the fifth metatarsal base. Through comparison and verification of the two, we describe the fracture line characteristics for avulsion fractures of the fifth metatarsal base and clarify the mechanisms of the ligaments and tendons in this fracture.

## Methods

### Inclusion and exclusion criteria

Participants inclusion criteria included: (i) age ≥ 18 years; (ii) avulsion fracture of the fifth metatarsal base, acute injury. Exclusion criteria included: (i) avulsion fracture of the fifth metatarsal base comorbid with other fractures of the foot; (ii) open fracture, pathologic fracture, or old fracture; (iii) foot deformity or variation (i.e. hallux valgus, talipes equinus), history of foot surgery involving tendons, ligaments or bony structures (i.e. tendon transposition, ligament repair or internal fixation for fractures).

### Type of study

This study is a retrospective audit of data already collected in the usual care of patients in our hospital. We retrospectively analysed the Computed Tomography (CT) scan data of 222 patients with the avulsion fractures of the fifth metatarsal base, who met the selection criteria above and were admitted to our hospital from August 2015 to August 2020. The patients included 97 females and 125 males, aged 18–80 years, with 117 affected on the right side and 105 on the left side (Table 1).

**Table 1:** Participant characteristics

	Number (percentage)
Participant (%)	222 (100.0)
Male	97 (43.8)
Female	125 (56.2)
Age, average (range), y	49.1 ± 14.33 (18–81)
Classification of fracture	
Ekrol^a^ I (%)	90 (40.5)
Ekrol^a^ II (%)	39 (17.6)
Ekrol^a^ III (%)	54 (24.3)
Special types (%)	39 (17.6)

^a^ Ekrol classified Lawrence zone I fractures into three types: type I: avulsion fractures of the tip of the tuberosity; type II: oblique fracture lines from the tuberosity to the fifth metatarso-cuboid joint; type III: transverse fractures just passing through the junction between the fourth and fifth metatarsal bases

Additionally, we selected 8 unpaired lower limbs of adult Asian frozen cadaveric specimens, with 4 on the left side and 4 on the right side. The specimens were of unknown age and gender and were randomly numbered from 1 to 8. Bone abnormalities such as osteoarticular degeneration, deformity, and tumour were excluded from the specimens using an X-ray. The articular surface area, tendon attachment area, and tendon diameter were measured with vernier calipers to an accuracy of 0.02 mm.

### Ethical approval

All patients signed the informed consent. This study was approved by the Institutional Ethics Committee of the authors’ institution (Shanghai Tongji Hospital, K-W-2020-018).

### Fracture line mapping and fracture surface heatmapping

**Fig. 1:**
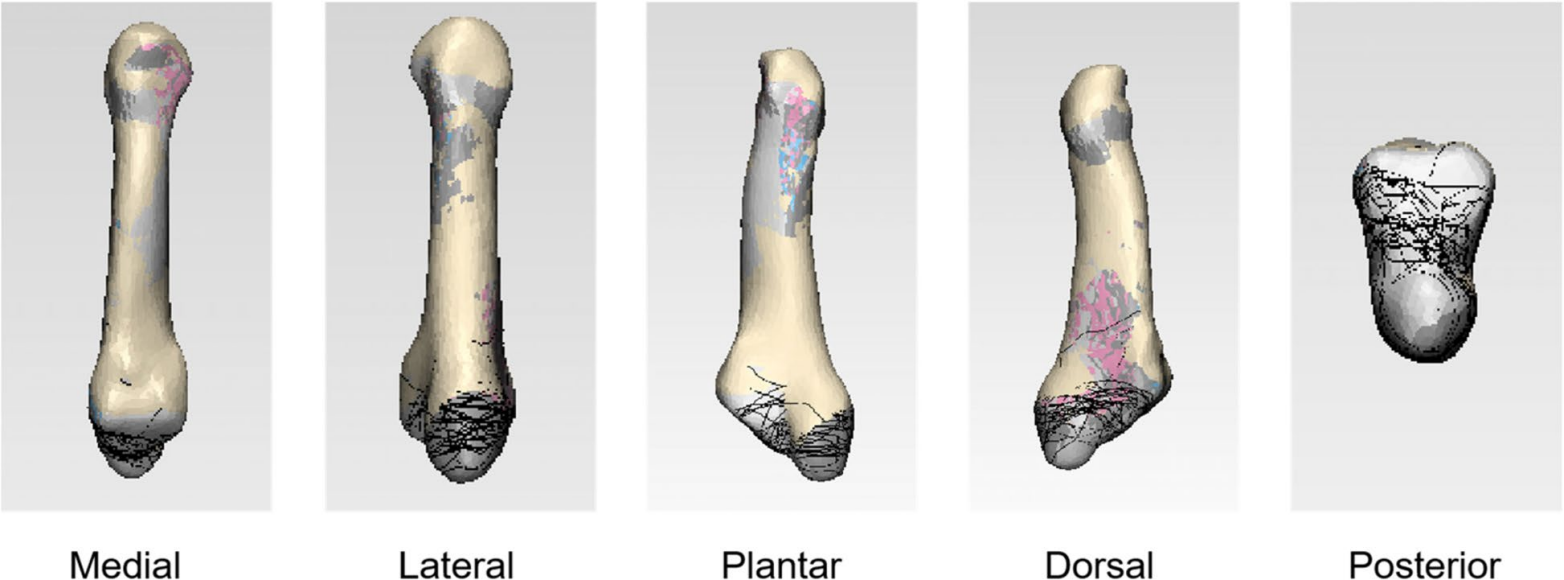
Representative views of the 3-D map of the 222 avulsion fractures of the fifth metatarsal base lines (left fifth metatarsal model)

**Fig. 2:**
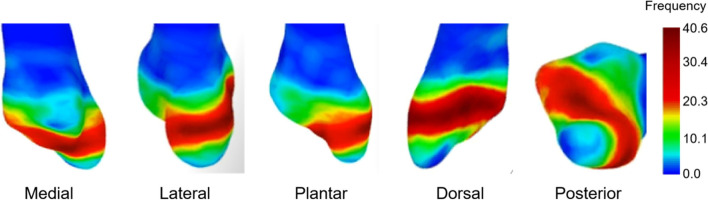
Representative views of the 222 avulsion fractures of the fifth metatarsal base lines heat map. The gradual change in color from red to blue indicates that the frequency of fracture lines changes from more to less

**Fig. 3:**
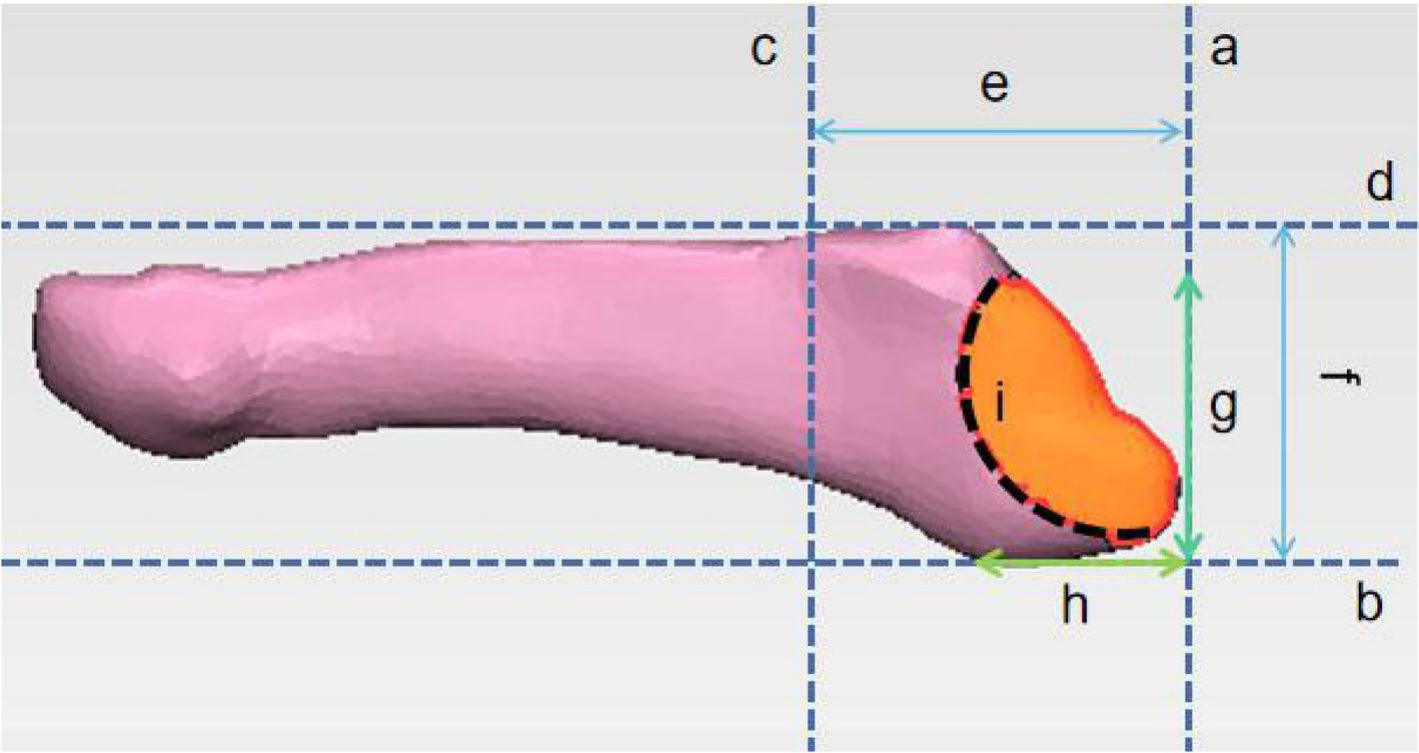
Schematic illustration of measurement and observation of distribution characteristics on the dorsal view. **a** Rear margin tangent; **b** Lateral margin tangent; **c** Connection of the medial and lateral attachment points of the fifth metatarsal metaphysis; **d** Medial margin tangent; **e** Anteroposterior diameter of the base of the fifth metatarsal bone; **f** Transverse diameter of the base of the fifth metatarsal bone; **g** Distance between fracture line and tangent line of lateral margin; **h** Distance between fracture line and tangent line of posterior margin; **i** Fracture line

A 64-slice, spiral computed graphic scanner was used in this study and the scanning parameters were 120 kV, 350 mA, 0.625 mm thickness, and 1-second rotation time. The patients’ CT data were exported in Joint Photographic Experts Group (JPEG) format, and 3D reconstruction was performed on the exported data using the Mimics 17.0 (Materialise NV) to obtain a stereoscopic image for the bony structure of the fifth metatarsal only. The fracture lines of the 222 patients were separately marked in 222 normalized 3D model of the fifth metatarsal bone first and then compiled them in one normalized 3D model to form a fracture lines map (Fig. 1). The compiled fracture lines were converted into a 3D heat map by using the E-3D digital medical platform (Central South University, Changsha, China). The conversion was based on the number of times the fracture line of the fifth metatarsal base avulsion fracture occurred at each location on the 3D image (Fig. 2). Measurement and observation of distribution characteristics were mainly observed on the dorsal view. Tangents were drawn on the lateral and posterior borders on the dorsal view of the standard fifth metatarsal bone, and a straight line was drawn along the medial and lateral attachment points of the fifth metatarsal metaphysis (close to the anterior edge of the fourth and fifth metatarsal articulations) to obtain the area of the fifth metatarsal base (Fig. 3a, b, c, d). The distances between the ends of the fracture line and the tangents on each side (Fig. 3g, h) and the ratio of the anteroposterior to the transverse diameters of the fifth metatarsal base (Fig. 3g/f, h/e) were described to locate it on the dorsal view. The 3D distribution of the fracture lines for the avulsion fracture of the fifth metatarsal base were observed to understand concentrated areas in the 3D distribution of fracture lines, which were summarized and classified.

### Anatomical dissection and measurements

A longitudinal incision was made on the skin of the lateral aspect of the fifth metatarsal bone. The incision was first extended proximally to identify the peroneus tertius and peroneus brevis, then distally to carefully separate the soft tissue, and free the peroneus tertius and peroneus brevis. The incision was then extended to the plantar aspect to identify and free the lateral band of the plantar fascia. Subsequently, the fifth metatarsal base was further separated from the cuboid bone and the fourth metatarsal base, then the intact fifth metatarsal bone, and the attached lateral band of the plantar fascia, peroneus brevis, and peroneus tertius were completely freed from the cadaveric specimen. The attachment location, shape, area, and tendon diameter of the fifth metatarsal bone, peroneus brevis, lateral band of the plantar fascia and peroneus tertius to the fifth metatarsal base were observed and measured with a vernier caliper. Tendon diameter measurements were taken 1 cm proximal to the tendon terminus at the fifth metatarsal bone to indicate tendon strength.

## Results

**Fig. 4:**
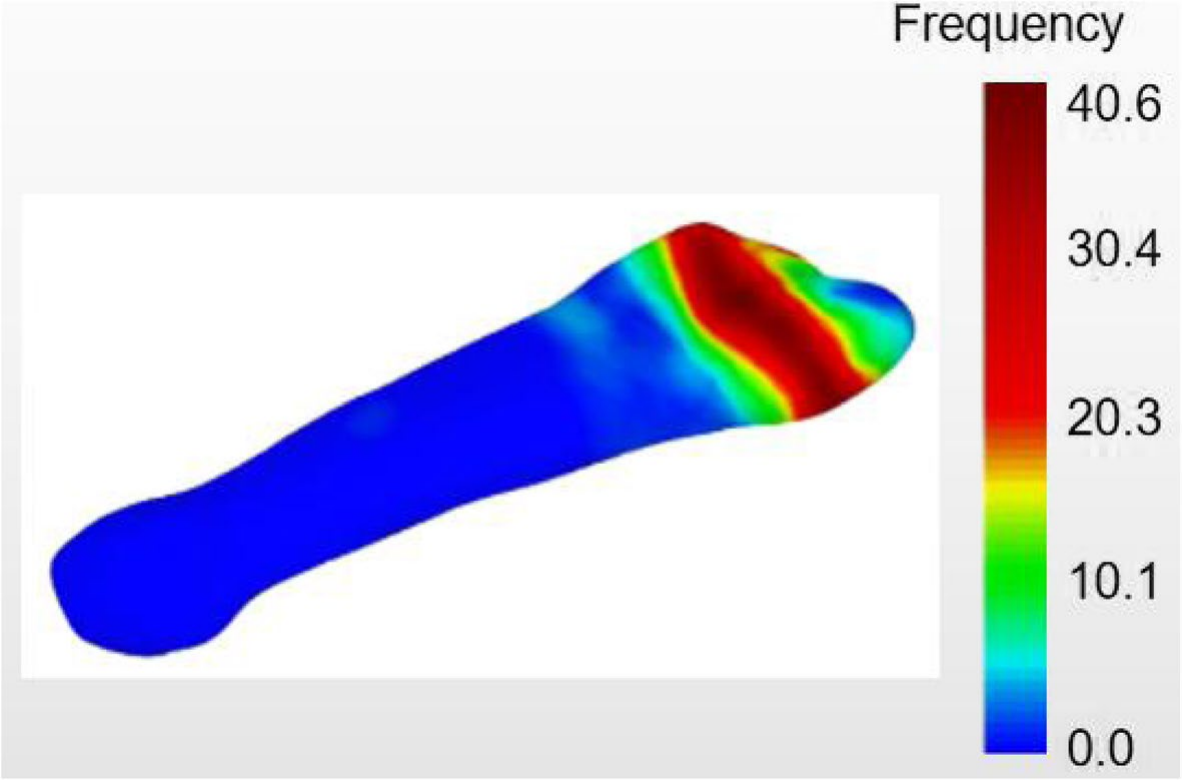
Dorsal view of the 222 avulsion fractures of the fifth metatarsal base lines heat map

Two authors (WH and BL) carried out all measurements and the average of these measurements were used. The fracture lines for the avulsion fractures of the fifth metatarsal base were varied but mostly concentrated in an arc-shaped band on the dorsal view. The band begun at 1/4 ~ 4/5 from the posterior tangent, with the start of the fracture line immediately adjacent to the lateral tangent and the end almost immediately adjacent to the medial tangent. The area of fracture lines with the highest frequency begun 2/5 from the posterior tangent and ended 7/10 from the posterior tangent (Fig. 4).

**Fig. 5:**
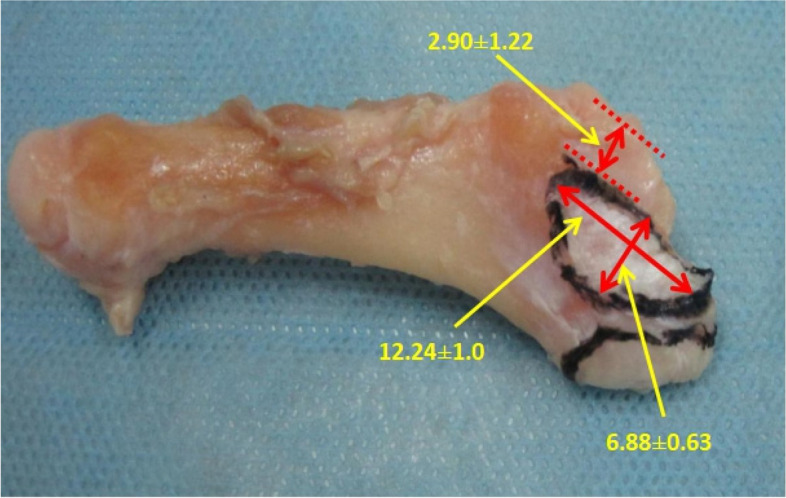
Base of the fifth metatarsal showing the attachment area of the peroneus brevis as it attaches on the dorsal metatarsal

**Fig. 6:**
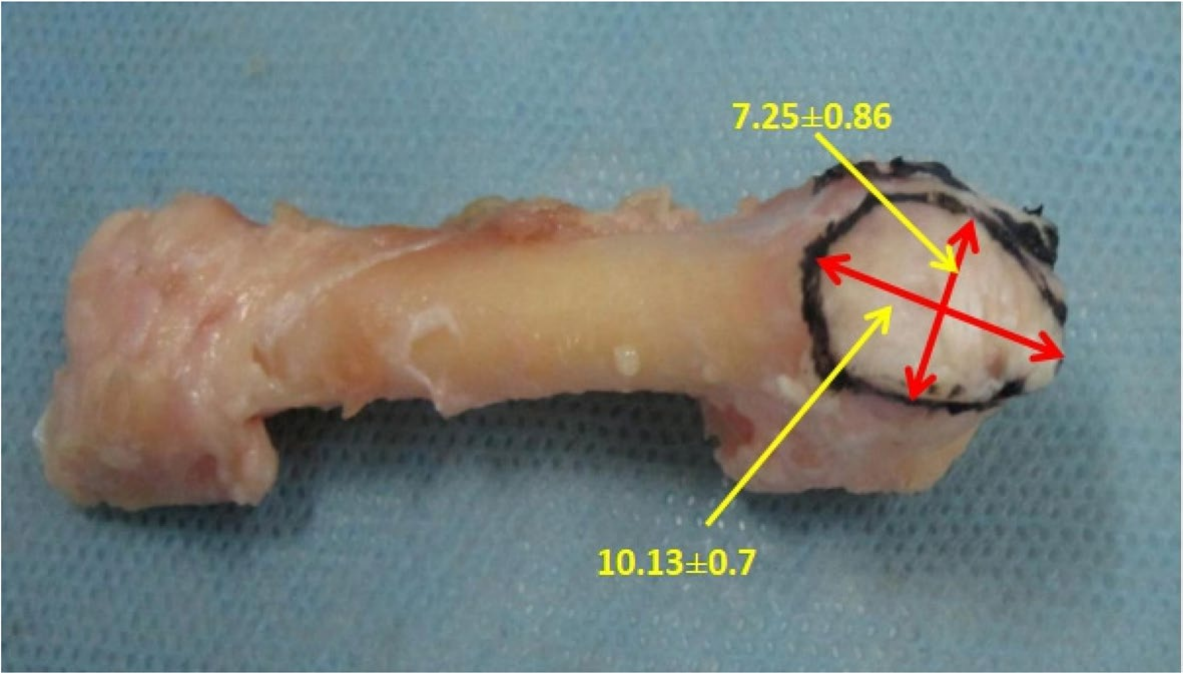
Base of the fifth metatarsal showing the attachment area of the lateral band of the plantar fascia as it attaches on the tuberosity

Eight adult foot specimens were observed and measured (Figs. 5 and 6). The posterior of the fifth metatarsal base was triangular in shape, and the medial aspect was the articular surface of the fourth and fifth metatarsals, which had a width of 9.59 ± 0.56 mm and a height of 10.90 ± 1.26 mm. The posterior plane consisted of the tarsometatarsal articular and non-articular surfaces of the fifth metatarsal bone, of which the non-articular surface had a width of 8.33 ± 0.70 mm and a height of 11.62 ± 1.18 mm, and the tarsometatarsal articular surface had a width of 15.14 ± 2.01 mm and a height of 13.83 ± 1.51 mm. Three main tendons or fascia were attached, namely peroneus brevis, lateral band of plantar fascia, and peroneus tertius. The peroneus brevis was attached to the dorsolateral aspect of the fifth metatarsal base, with a tendon diameter of 5.70 ± 0.37 mm; on the dorsal view of the fifth metatarsal bone, it was oval shaped and had an attachment area to the fifth metatarsal bone with long diameter 12.24 ± 1.09 mm and short diameter 6.88 ± 0.63 mm; its proximal edge was adjacent to the edge of the fifth metatarsal tarsometatarsal articular surface, its lateral edge was adjacent to the edge of the lateral band of the plantar fascia, and the shortest distance of its medial edge to the fourth and fifth metatarsal articular surface was 2.90 ± 1.22 mm. The lateral band of the plantar fascia was adjacent to the lateral edge of the peroneus brevis and encircled the fifth metatarsal tuberosity, with a tendon diameter of 6.65 ± 0.54 mm; on the lateral view of the fifth metatarsal bone, it was oval shaped and had an attachment area with a width of 10.13 ± 0.77 mm and a height of 7.25 ± 0.86 mm. The peroneus tertius was attached at the distal dorsal end of the fifth metatarsal base, with a smaller, oval-shaped attachment area and a tendon diameter of 3.31 ± 0.73 mm; the peroneus tertius was absent in one of the specimens (Tables 2, 3, 4 and 5). The anatomical measurements collected were analysed using Microsoft Excel 2013 (Microsoft Corporation, Redmond, Washington USA).

**Table 2:** Morphology of the fifth metatarsal

No.	Nonarticular Height (mm)	Nonarticular Width (mm)	Tarsometatarsal Articular Surface Width (mm)	Tarsometatarsal Articular Surface Height (mm)	Medial Articular Surface Width (mm)	Medial Articular Surface Height (mm)	Shape
1	13.24	7.14	11.52	13.64	9.24	9.42	Triangular
2	11.68	9.22	13.52	11.02	9.22	9.42	Triangular
3	10.02	8.02	17.56	13.72	9.12	12.86	Triangular
4	10.04	8.66	16.82	15.42	9.58	10.58	Triangular
5	11.36	8.88	15.56	14.22	9.84	11.22	Triangular
6	12.28	8.64	16.88	15.66	9.04	10.02	Triangular
7	12.86	8.52	14.98	12.54	10.68	12.06	Triangular
8	11.44	7.58	14.28	14.38	9.98	11.64	Triangular
Average	11.62	8.33	15.14	13.83	9.59	10.90	
SD	1.18	0.70	2.01	1.51	0.56	1.26

**Table 3:** Plantar fascia

No.	Width^a^ (mm)	Height^a^ (mm)	Tendon diameter (mm)	Shape^a^
1	9.22	8.22	7.46	Oval
2	9.04	7.02	6.68	Oval
3	10.92	6.02	5.92	Oval
4	9.58	8.32	6.38	Oval
5	9.98	7.36	6.72	Oval
6	10.86	7.68	5.98	Oval
7	10.74	7.22	7.02	Oval
8	10.66	6.12	7.04	Oval
Average	10.13	7.25	6.65	
SD	0.77	0.86	0.54	

^a^These data were measured where the plantar fascia attached to the fifth metatarsal bone surface

**Table 4:** Peroneus brevis

No.	Long diameter^a^ (mm)	Short diameter^a^ (mm)	Medial Border to Medial Metatarsal^a^ (mm)	Tendon Diameter (mm)	Shape^a^
1	10.02	6.12	1.50	5.98	Oval
2	12.02	7.02	1.50	6.04	Oval
3	13.74	8.02	3.82	6.12	Oval
4	12.82	6.46	4.00	5.82	Oval
5	12.88	6.16	2.68	5.62	Oval
6	12.02	6.88	4.86	5.02	Oval
7	12.58	7.14	2.40	5.46	Oval
8	11.86	7.22	2.42	5.52	Oval
Average	12.24	6.88	2.90	5.70	
SD	1.09	0.63	1.22	0.37	

^a^These data were measured where the peroneus brevis attached to the fifth metatarsal bone surface

**Table 5:** Peroneus tertius

NO.	Shape^a^	Tendon diameter (mm)
1	Oval	2.12
2	Oval	3.02
3	Oval	3.16
4	Oval	4.02
5	Oval	3.84
6	Oval	4.14
7	Oval	2.88
8		Absent
Average		3.31
SD		0.73

^a^This data was measured where the peroneus tertius attached to the fifth metatarsal bone surface

## Discussion

To our knowledge, this is the first study to elucidate the avulsion fractures of the fifth metatarsal base by means of 3D fracture mapping techniques in combination with anatomical research.

Several anatomical and radiographic studies have been conducted on the fifth metatarsal base. Imre et al. [5] used magnetic resonance imaging and anatomical dissection to study differences in the attachment site of the peroneus brevis to the fifth metatarsal base and have categorized it into six attachment types and mentioned that a narrowly inserted tendon may apply more stress since the internal force applied per unit area is increased when compared with a wider insertion area. Increased stress may eventually lead to a higher tension and may result in an increased risk of fracture. DeVries et al. [8] performed an anatomical studies on 10 frozen cadaveric specimens, where the attachment sites of the peroneus brevis and the lateral band of the plantar fascia at the fifth metatarsal base was defined as the boundary to divide the tuberosity of the fifth metatarsal into three zones. Although the study noticed that different zones of fractures may be caused by peroneus brevis or the lateral band of the plantar fascia, no further research has been conducted to verify it and the description of the mechanism of injury for avulsion fractures of the fifth metatarsal base remains uncertain. Morris et al. [12] performed anatomical and biomechanical study of the effect of peroneus brevis on the stability of the base fracture of the fifth metatarsal bone showed that mechanical instability secondary to the deforming force of the peroneus brevis may play a contributory role in delayed union and nonunion of these fractures. Conversely, by spanning the fracture site of avulsion fractures, the peroneus brevis insertion may act to stabilize avulsion injuries in a tension band manner. Existing studies fail to properly describe the fracture characteristics in the avulsion fractures of the fifth metatarsal base and explain how they were produced because each study only used a single assessment technique, radiographic or anatomical, and failed to elucidate the mechanism of the fracture line formation.

**Fig. 7:**
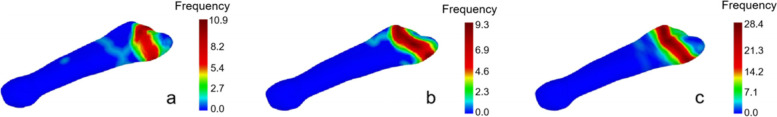
The fracture lines heat map on the dorsal view of fifth metatarsal base for different injury mechanisms. **a** Involves the action of the lateral band of the plantar fascia predominantly (48 ft); **b** Involves the action of the peroneus brevis predominantly (54 ft); **c** Involves the joint action of the peroneus brevis and the lateral band of the plantar fascia (120 ft)

Therefore, the present study improved the 3D heat mapping of fracture lines by previous studies [13–15] and conducted the 3D examination on the fracture line characteristics of 222 cases with the avulsion fractures of the fifth metatarsal base. The fracture lines heat map on the dorsal view of fifth metatarsal base could be divided into three zones (bounded by red bands). The distal and proximal fracture lines rarely pass through the tarsometatarsal articular surface, while the middle fracture line often passes through the tarsometatarsal articular surface and sometimes through both cortices without involving the articular surface. However, the fracture lines rarely involve the articulation on the lateral aspect of the 4th metatarsal. In addition, for the anatomical study, the lateral band of the plantar fascia and peroneus brevis are attached to the dorsolateral aspect of the fifth metatarsal base. Since the attachment point of the peroneus tertius is farther away from the fifth metatarsal base and the peroneus tertius has less strength than the other two tendons [16], we propose that the peroneus tertius does not play a major role in the avulsion fracture of the fifth metatarsal base. Following this, separate heat maps were generated for the fracture lines in the three zones that appeared most frequently on the overall heat map. Combined with the anatomical study, we found that the avulsion fragment coincided with the area containing the lateral band of the plantar fascia and peroneus brevis (Fig. 7). Therefore, we hypothesise that the lateral band of the plantar fascia and peroneus brevis play a major role in the fifth metatarsal base avulsion fracture together or separately, which leads to fracture lines concentrating in three zones.

There are several clinical classifications for the avulsion fractures of the fifth metatarsal base. Ekrol et al. [17] classified Lawrence zone I fractures into three types: type I: avulsion fractures of the tip of the tuberosity; type II: oblique fracture lines from the tuberosity to the fifth metatarso-cuboid joint; type III: transverse fractures just passing through the junction between the fourth and fifth metatarsal bases. Mehlhorn et al. [18] developed a classification system based on an increased risk for secondary displacement of fractures with a more medial joint entry of the fracture line at the fifth metatarsal base. Type I, II, or III were defined dependent on the joint entry of the fracture line at the fifth metatarsal base (lateral one-third, middle one-third, and medial one-third). Fractures without displacement were summarized as A-type (I-IIIA) and with a fracture-step-off > 2 mm as B-type (I-IIIB). However, in our study, we found some cases with two fracture lines or across two zones, and even some fracture lines that were curved or folded. Various types of the avulsion fractures of the fifth metatarsal base can be found in clinical practice; for example, two fracture lines that intersect can exist simultaneously.

**Fig. 8:**
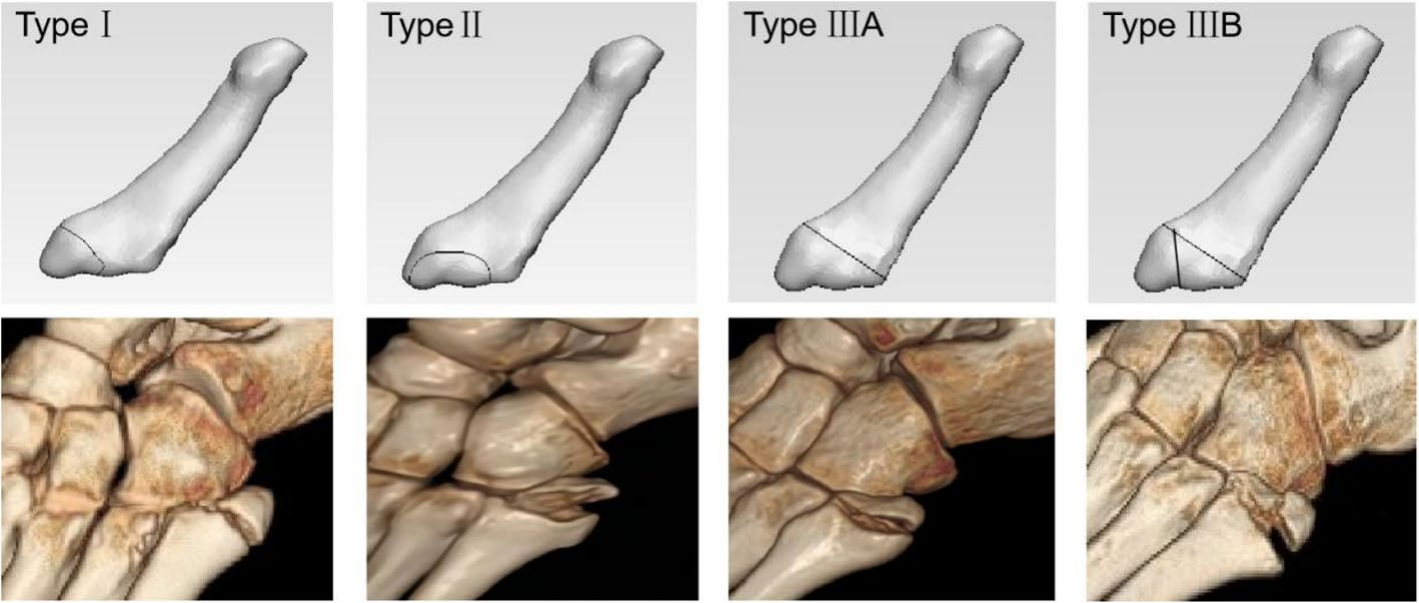
A classification system was developed based on injury mechanism and characterization of fracture lines. The image with gray background is the schematic diagram of fracture classification, and the image with black background below is the 3D reconstruction map of corresponding types of patients in this study: type I predominantly involves the action of the lateral band of the plantar fascia; type II predominantly involves the action of the peroneus brevis; type IIIA involves the joint action of the peroneus brevis and the lateral band of the plantar fascia, with only one fracture line; and type IIIB involves the joint action of the peroneus brevis muscle and the lateral band of the plantar fascia, with two fracture lines

The classifications above do not provide an adequate description for these special types of fracture lines and are insufficiently three-dimensional, so a more comprehensive and rational classification, which can help clinicians when planning treatment, is needed. Hence, we can use the imaging presentations of patients’ fracture lines to help distinguish which soft tissue insertion may be involved in the avulsion fracture and thus help to infer the mechanism of injury. Finally, we developed a classification based on fracture pattern and soft tissue insertion which can help guide the soft tissue involvement and potential injury mechanism (Fig. 8): proximal fracture lines (type I) predominantly involves the action of the lateral band of the plantar fascia; middle fracture lines (type II) predominantly involves the action of the peroneus brevis; distal fracture lines (type IIIA) involves the joint action of the peroneus brevis and the lateral band of the plantar fascia, with only one fracture line, and type IIIB involves the joint action of the peroneus brevis muscle and the lateral band of the plantar fascia, with two fracture lines. As this classification is based on injury mechanism, the new classification can still be used to make an correct categorization with full knowledge of the tendon characteristics and it can describe almost all types of the avulsion fractures of the fifth metatarsal base more accurately than existing classifications.

The treatments for the avulsion fractures of the fifth metatarsal base are also diverse [19]. Although Some studies [20–22] showed the majority of the fifth metatarsal base avulsion fractures are successfully treated non-operatively. Although it was not the aim of our study, we followed 30 conservatively treated patients for 3 months and 5 patients (all of them had type III fractures) experienced fracture redisplacement. Therefore, for fractures classified as type III with significantly displaced (≥2 mm), we still recommend surgical treatment. There are two main reasons. Firstly, the forces produced by both tendons may lead to the increased risk of redisplacement and non-healing. Also, articular surface injury occurred in almost all the fractures of type III in this study and conservative treatment may lead to intractable pain caused by traumatic arthritis. While more convincing follow-up studies with large samples are still needed to further determine the best treatment option. What is important is that, in addition, 10 patients in our study who were classified with a type I fracture actively requested surgical treatment, and on further examination, these patients also had a peroneus brevis rupture at the tip of the lateral malleolus. This may be due to the fact that the force from peroneus brevis was buffered by its self-rupture. So, for patients with type I fractures, doctors should consciously check the integrity of peroneus brevis using Magnetic Resonance Imaging (MRI) or ultrasound.

Although 222 patients were enrolled in this study and this study is the first to combine 3D CT imaging and anatomical assessment to investigate the mechanism of injury in avulsion fractures of the fifth metatarsal base, the sample size of specimens used for autopsy was small. As patients and specimens in the study are from our country only, and we did not undertake reliability testing of the procedures/measurements undertaken, further clinical application and biomechanical studies are required to demonstrate the validity and reliability of this classification.

## Conclusion

In conclusion, the avulsion fractures of the fifth metatarsal base produce various fracture lines. By examining their 3D distribution and anatomical features, this study has suggested that the lateral band of the plantar fascia and peroneus brevis play a major role in avulsion fractures of the fifth metatarsal base. Trauma to these structures can occur separately or together, so we have proposed a novel classification for such fractures, which can serve as a reference for clinical treatment and diagnosis.

## Data Availability

The datasets generated during and/or analyzed during the current study are available from the corresponding author on reasonable request.

## Abbreviations

3D: 3-dimensional
CT: Computed Tomography
JPEG: Joint Photographic Experts Group
MRI: Magnetic Resonance Imaging
